# PRONTOX – proton therapy to reduce acute normal tissue toxicity in locally advanced non-small-cell lung carcinomas (NSCLC): study protocol for a randomised controlled trial

**DOI:** 10.1186/s13063-016-1679-4

**Published:** 2016-11-15

**Authors:** Sebastian Zschaeck, Monique Simon, Steffen Löck, Esther G. C. Troost, Kristin Stützer, Patrick Wohlfahrt, Steffen Appold, Sebastian Makocki, Rebecca Bütof, Christian Richter, Michael Baumann, Mechthild Krause

**Affiliations:** 1 Department of Radiation Oncology, Faculty of Medicine and University Hospital Carl Gustav Carus, Technische Universität Dresden, OncoRay – National Center for Radiation Research in Oncology, Faculty of Medicine and University Hospital Carl Gustav Carus, Technische Universität Dresden and Helmholtz-Zentrum Dresden – Rossendorf, German Cancer Consortium (DKTK) Dresden and German Cancer Research Center (DKFZ), National Center for Tumor Diseases (NCT), Partner Site Dresden, Fetscherstr. 74, Dresden, 01307 Germany; 2 German Cancer Consortium (DKTK), German Cancer Research Center (DKFZ), Department of Radiation Oncology, Faculty of Medicine and University Hospital Carl Gustav Carus, Technische Universität Dresden, National Center for Tumor Diseases (NCT), Partner Site Dresden, Fetscherstr. 74, Dresden, 01307 Germany; 3 OncoRay – National Center for Radiation Research in Oncology, Faculty of Medicine and University Hospital Carl Gustav Carus, Technische Universität Dresden and Helmholtz-Zentrum Dresden – Rossendorf, National Center for Tumor Diseases (NCT), Partner Site Dresden, Fetscherstr. 74, Dresden, 01307 Germany; 4 Helmholtz-Zentrum Dresden – Rossendorf, Institute of Radiooncology, Department of Radiation Oncology, Faculty of Medicine and University Hospital Carl Gustav Carus, Technische Universität Dresden, OncoRay – National Center for Radiation Research in Oncology, German Cancer Consortium (DKTK), German Cancer Research Center (DKFZ), National Center for Tumor Diseases (NCT), Partner Site Dresden, Bautzner Landstr. 400, Dresden, 01328 Germany; 5 OncoRay – National Center for Radiation Research in Oncology, Faculty of Medicine and University Hospital Carl Gustav Carus, Technische Universität Dresden, Helmholtz-Zentrum Dresden – Rossendorf, Fetscherstr. 74, PF 41, Dresden, 01307 Germany; 6 Department of Radiation Oncology, Faculty of Medicine and University Hospital Carl Gustav Carus, Technische Universität Dresden, National Center for Tumor Diseases (NCT), Partner Site Dresden, Fetscherstr. 74, Dresden, 01307 Germany; 7 Department of Radiation Oncology, Faculty of Medicine and University Hospital Carl Gustav Carus, Technische Universität Dresden, OncoRay – National Center for Radiation Research in Oncology, Faculty of Medicine and University Hospital Carl Gustav Carus, Technische Universität Dresden and Helmholtz-Zentrum Dresden – Rossendorf, National Center for Tumor Diseases (NCT), Partner Site Dresden, Fetscherstr. 74, Dresden, 01307 Germany; 8 OncoRay – National Center for Radiation Research in Oncology, Faculty of Medicine and University Hospital Carl Gustav Carus and Helmholtz-Zentrum Dresden – Rossendorf, Technische Universität Dresden,German Cancer Consortium (DKTK) Dresden and German Cancer Research Center (DKFZ), Heidelberg, Helmholtz-Zentrum Dresden – Rossendorf, Institute of Radiooncology, Fetscherstr. 74, Dresden, 01307 Germany; 9 Department of Radiation Oncology, Faculty of Medicine and University Hospital Carl Gustav Carus, Technische Universität Dresden, OncoRay – National Center for Radiation Research in Oncology, Faculty of Medicine and University Hospital Carl Gustav Carus, Technische Universität Dresden and Helmholtz-Zentrum Dresden – Rossendorf, German Cancer Consortium (DKTK) Dresden and German Cancer Research Center (DKFZ) Heidelberg, Helmholtz-Zentrum Dresden – Rossendorf, Institute of Radiooncology, National Center for Tumor Diseases (NCT), Partner Site Dresden, Fetscherstr. 74, Dresden, 01307 Germany; 10 Department of Radiation Oncology, Faculty of Medicine and University Hospital Carl Gustav Carus, Technische Universität Dresden, OncoRay – National Center for Radiation Research in Oncology, Faculty of Medicine and University Hospital Carl Gustav Carus, Technische Universität Dresden and Helmholtz-Zentrum Dresden – Rossendorf German Cancer Consortium (DKTK) Dresden, German Cancer Research Center (DKFZ) Heidelberg, Helmholtz-Zentrum Dresden – Rossendorf, National Center for Tumor Diseases (NCT), Partner Site Dresden, Fetscherstr. 74, Dresden, 01307 Germany

**Keywords:** Proton radiotherapy, Non-small-cell lung cancer (NSCLC), Locally advanced, Photon radiotherapy, Toxicity, Randomised clinical trial, Phase II trial

## Abstract

**Background:**

Primary radiochemotherapy with photons is the standard treatment for locally advanced-stage non-small cell lung cancer (NSCLC) patients. Acute radiation-induced side effects such as oesophagitis and radiation pneumonitis limit patients’ quality of life, and the latter can be potentially life-threatening. Due to its distinct physical characteristics, proton therapy enables better sparing of normal tissues, which is supposed to translate into a reduction of radiation-induced side effects.

**Methods/design:**

This is a single-centre, prospective, randomised controlled, phase II clinical trial to compare photon to proton radiotherapy up to 66 Gy (RBE) with concomitant standard chemotherapy in patients with locally advanced-stage NSCLC. Patients will be allocated in a 1:1 ratio to photon or proton therapy, and treatment will be delivered slightly accelerated with six fractions of 2 Gy (RBE) per week.

**Discussion:**

The overall aim of the study is to show a decrease of early and intermediate radiation-induced toxicity using proton therapy. For the primary endpoint of the study we postulate a decrease of radiation-induced side effects (oesophagitis and pneumonitis grade II or higher) from 39 to 12%. Secondary endpoints are locoregional and distant failure, overall survival and late side effects.

**Trial registration:**

Registered at ClinicalTrials.gov with Identifier NCT02731001 on 1 April 2016.

**Electronic supplementary material:**

The online version of this article (doi:10.1186/s13063-016-1679-4) contains supplementary material, which is available to authorized users.

## Background

Primary lung tumours are a leading cause for tumour-related mortality worldwide, which is mainly due to their unfavourable prognosis, particularly in the advanced or metastasised stages. Non-small cell lung cancer (NSCLC) accounts for the vast majority of primary lung cancers. The treatment of choice for inoperable or locally advanced NSCLC without distant metastasis is primary radiochemotherapy [1]. The prescribed standard radiation dose ranges between 60 and 66 Gy in 2-Gy fractions, delivered using three-dimensional conformal or intensity-modulated radiation therapy (3D-CRT and IMRT, respectively). As local failure occurs in about 50% of patients with locally advanced disease, current approaches aim for escalation of radiation dose. The phase III RTOG 0617 trial randomised patients to 60 Gy standard dose or 74 Gy dose escalation after promising phase I and II trials [2]. Unexpectedly, median overall survival of patients in the high-dose group was decreased compared to standard fractionation. The underlying reasons are currently being assessed and may include insufficient target coverage and increased dose to the heart.

In general, pulmonary, oesophageal and cardiac toxicity [3] constitute the most relevant acute and late toxicities after thoracic radiotherapy. Therefore, future studies on improved radiation treatment for NSCLC have to include vigorous reduction of normal tissue complication probabilities. One promising approach is the use of proton beam radiotherapy. Proton therapy offers advantages in normal tissue sparing due to the sharp dose fall-off behind the target volume covered by the spread-out Bragg peak that it produces. In a retrospective analysis, patients with advanced stages of NSCLC treated with proton beam therapy presented with fewer radiation-induced side effects although they were treated with 74 Gy (RBE) (= photon equivalent dose by considering an average relative biological effectiveness (RBE) of 1.1) compared to the photon control group which had a prescribed dose of only 63 Gy [4]. Proton therapy offers unique opportunities for dose escalation in NSCLC due to its sparing of low and intermediate radiation doses to surrounding normal tissues. Before applying dose escalation with proton beam therapy, prospective studies confirming lower normal tissue toxicity for proton therapy should be performed to ensure maximum patient safety.

The aims of the present study are to demonstrate a reduction of acute radiation-induced side effects, i.e. pneumonitis and oesophagitis grade II or higher, using proton versus photon radiochemotherapy to equal radiation doses. Secondary endpoints include evaluation of quality of life, locoregional control, occurrence of distant metastases, survival and late radiation-induced side effects.

## Methods/design

This is a single-centre, prospective, randomised controlled, phase II clinical trial comparing slightly accelerated radiochemotherapy with photons to that with protons in patients with inoperable or locally advanced, cytologically or histologically confirmed NSCLC. Dose prescription to the tumour is 66 Gy (RBE), maximal doses to the organs at risk comply with international standards. All patients undergo complete clinical staging with Fluorodeoxyglucose-Positron Emission Tomography-Computer Tomography (FDG-PET-CT) and magnetic resonance imaging (MRI) of the brain.

### Inclusion criteria

- NSCLC (confirmed by cytology or histology) staged UICC IIIA or IIIB or UICC II if the tumour is medically inoperable or the patient declines surgery
- No distant metastases (M1)
- Patient is aged between 18 and 70 years
- Patient is medically suited for primary radiochemotherapy with curative intent
- Patient has signed a declaration of informed consent
- Adequate compliance for treatment and clinical follow-up
- Adequate contraception during and after therapy if indicated

### Exclusion criteria

- Participation in another interventional trial at the same time
- T1 or T2 N0 tumours that are suitable for stereotactic radiotherapy
- Relevant neurological or psychiatric disorders that hinder treatment, follow-up or understanding of the procedures
- Pregnant or breastfeeding women
- Prior thoracic radiotherapy
- History of other malignancies during the last 5 years (exceptions can be made for tumours with excellent outcome)
- Unintended weight loss greater than 15% before therapy
- Serological alterations (liver, kidney) prohibiting application of simultaneous chemotherapy
- Respiratory motion of the tumour of more than 10 mm (evaluated by four-dimensional computer tomography (4D-CT), also when methods for motion reduction (abdominal compression) are applied

### Recruitment, randomisation and workflow

**Fig. 1:**
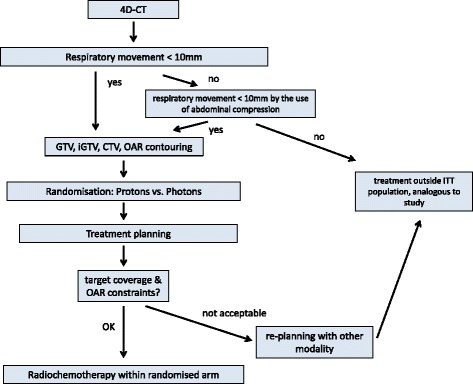
Flowchart of planning procedures and patient allocation

To assess whether patients can be treated with protons, a 4D-CT will be performed to evaluate the respiratory motion of the tumour. If tumour motion is larger than 10 mm, abdominal compression or gating methods can be used and the patient will undergo a second 4D-CT scan. If the respiratory motion is still too large, the patient will be treated with photons within an observational cohort. For patients with tumour motion of less than 10 mm, the gross tumour volume (GTV), clinical target volume (CTV) and organs at risk (OAR) will be delineated. Patients are then randomised for proton or photon therapy and the respective radiation plans will be calculated. If target coverage is insufficient or doses at OAR are too high, a new treatment plan using the other modality (photons or protons respectively) will be calculated. If the other plan adheres better to the constraints, the patient is treated with the respective modality within an observational cohort. Patient reallocation to another treatment arm due to nonconformity to the constraints will be statistically handled as an event in the randomised (intent-to-treat) arm. This procedure ensures maximum patient safety while not compromising results for randomised patients. Figure 1 shows a flowchart of patient allocation to treatment arms.

### Radiotherapy

Treatment is planned using FDG-PET-CT in the treatment position and an additional 4D-CT to assess tumour motion. On the basis of the 4D-CT an internal gross tumour volume (iGTV) is generated that encompasses the whole tumour motion during respiration. The CTV is generated by summing the iGTVs. Patients within the photon arm will be treated using IMRT with six fractions per week to a total dose of 66 Gy. Patients within the proton arm will receive 66 Gy (RBE) also delivered with six fractions per week. Target coverage and dose to organs at risk will be assessed by at least one physician and one physicist. Constraints will be according to current clinical guidelines in combination with the RTOG 1308 constraints, whichever is stricter than the QUANTEC criteria [5]. During the course of radiotherapy in-room control 4D-CTs (maximum two per week) will be acquired to check for stability of inner anatomy and motion characteristics.

**Fig. 2:**
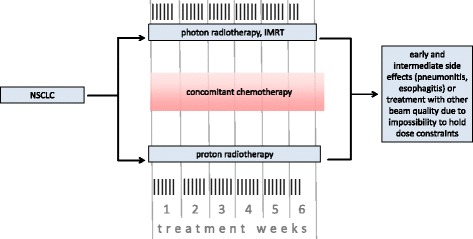
Flowchart of both randomised treatment arms and primary endpoint

Chemotherapy will be delivered according to current clinical standards without differences for both groups. Figure 2 shows the planned workflow for both treatment arms.

### Primary and secondary endpoints

The primary aim of the study is to show whether a reduction of acute and intermediate (i.e. late-occurring pneumonitis) radiation-induced side effects (pneumonitis and dysphagia grade II or higher scored by Common Terminology Criteria for Adverse Events CTC-AE 4.0) can be achieved using proton as compared to photon beam therapy. The primary endpoint of the study will be the occurrence of radiation-induced side effects (CTC-AE 4.0 scoring) up to 6 months after treatment or re-planning with another radiation modality due to infringement of OAR or tumour coverage constraints. Secondary endpoints contain classification of severity of early and late side effects in both groups, comparison of quality of life and of treatment outcome; furthermore, dosimetric parameters and CT alterations of normal tissues at follow-up will be compared between both groups. Quality of life will be assessed additionally by the European Organisation for Research and Treatment of Cancer (EORTC) questionnaires C30 and LC13 before and at the end of treatment and at each follow-up visit. For follow-up, patients will be interviewed via telephone 2, 4, 8 and 10 weeks after treatment based on a standardised form. Clinical follow-up examinations with additional CT or FDG-PET-CT scans are scheduled 6 weeks, 3 months and 6 months after treatment. After that, follow-up will be continued outside the trial. During therapy and at follow-up, scoring of side effects will be performed according to CTC-AE 4.0. After that, follow-up will be continued outside the trial and scoring of side effects will also be performed according to CTC-AE 4.0.

The trial design and protocol adhere to Standard Protocol items: Recommendations for Interventional Trials (SPIRIT) criteria (www.spirit-statement.org); the SPIRIT checklist and figure can be found as Additional file 1: Table S1 and Additional file 2: Figure S1.

## Statistics

The primary endpoint of the study is the occurrence of acute and intermediate radiation-induced side effects, which will be observed in randomised patient cohorts treated with photon or proton therapy in a 1:1 ratio. For photon therapy, the incidence of pneumonitis and dysphagia grade II or higher at our institution is approximately 18% and 25%, respectively, which compares favourably with incidences in the published literature [6, 7]. Using proton therapy, a reduction by a factor of 4–5 seems achievable [5]. Therefore, conservative estimates for pneumonitis and dysphagia occurrence are 4% and 8%, respectively. Assuming that the two side effects are independent, the combined fraction of expected side effects is 0.18 + 0.25 – 0.18 × 0.25 = 0.39 (39%) for photon therapy and 0.04 + 0.08 – 0.04 × 0.08 = 0.12 (12%) for proton therapy.

Using these values, the required patient number to reveal a significant difference in the occurrence of side effects between the two arms is calculated as 39. This number is based on a one-sided test of proportions with a continuity correction using a normal approximation (STATA 11.2, StataCorp LP, College Station, TX, USA, function sampsi). The significance level was set to 0.05 and the power to 0.8. A one-sided test seems appropriate since a reduced occurrence of side effects is expected from proton treatment. Assuming a 20% patient dropout the final number of patients per treatment arm is 49.

## Discussion

**Fig. 3:**
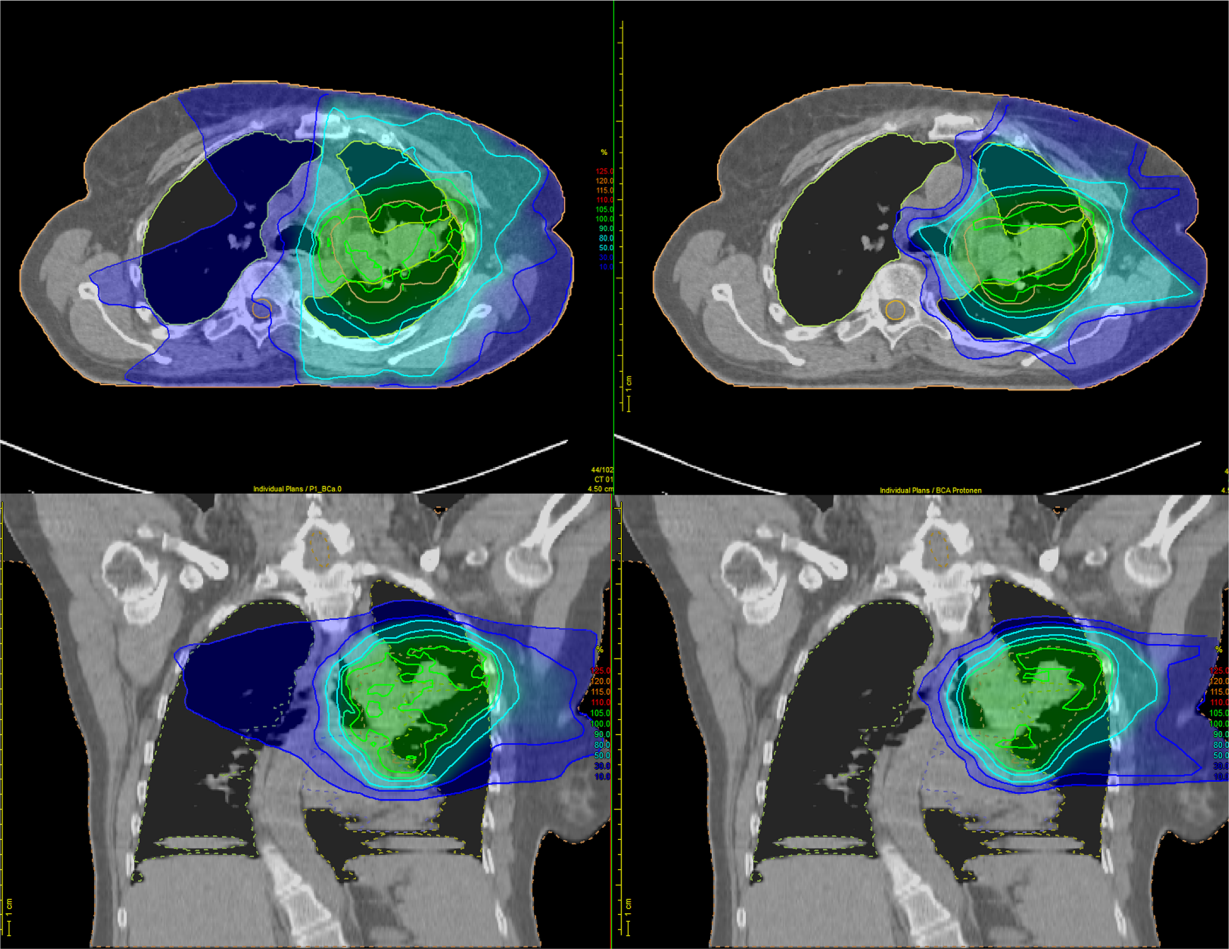
Dose distribution of one patient with locally advanced non-small cell lung cancer (NSCLC) planned with intensity-modulated radiation therapy (IMRT) (left) or protons (right) showing lower doses to organs at risk (OAR) by proton therapy

The aim of this prospective randomised study is to evaluate whether radiochemotherapy for advanced-stage NSCLC patients with protons leads to decreased radiation-induced side effects as compared to photons. Acute side effects are of particular importance in thoracic tumour sites as compared to other radiation sites. Acute radiation toxicity is expected to decrease quality of life of cancer patients, though mostly only temporarily, and thus is currently being subordinated to potential tumour control in a curative setting. Radiation-induced pneumonitis, however, is a potentially lethal toxicity and can develop into chronic fibrosis, often causing deteriorating in patients’ long-time quality of life [8]. Exposure of the lung, and probably also the heart, to low and intermediate radiation doses is highly relevant for the development of pneumonitis and can be reduced by proton therapy (see Fig. 3 for dose distributions for IMRT and proton radiotherapy) [7]. Although there is a strong correlation between radiation dose and local control in preclinical studies and in studies using hypofractionated stereotactic radiotherapy or alternative treatment modification, in advanced disease dose escalation with photons did not translate into a survival benefit in the recently published RTOG 0617 study [2, 9–13]. It is noteworthy that in the latter study the dose escalation was performed without acceleration, even though a time factor exists for locally advanced NSCLC as confirmed by the CHARTWEL study [14]. Protons, by a better sparing of surrounding healthy tissue, may be able to increase the dose to the tumour without an increase in toxicity [15]. If the present study demonstrates a decrease in toxicity for equal-dose radiotherapy allied with protons versus photons, increasing radiation doses with protons within a clinical trial would be the next step to achieve better local tumour control, which may then also be related to increased overall survival and metastasis-free survival as shown by others [16].

## Trial status

The trial is currently recruiting.

## Additional files

Additional file 1: Table S1. SPIRIT 2013 checklist PRONTOX. (DOC 121 kb)
Additional file 2: Figure S1. SPIRIT 2013 figure PRONTOX. (DOC 51 kb)

## Abbreviations

3D-CRT: three-dimensional conformal radiation therapy
4D-CT: Four-dimensional computer tomography
CT: Computer tomography
CTC-AE: Common Terminology Criteria for Adverse Events
CTV: Clinical target volume
FDG: Fluorodeoxyglucose
GTV: Gross tumour volume
Gy: Gray
iGTV: Internal gross tumour volume
IMRT: Intensity-modulated radiation therapy
MRI: Magnetic resonance imaging
NSCL: Non-small-cell lung carcinoma
OAR: Organs at risk
PET: Positron Emission Tomography
RBE: Relative biological effectiveness
