# Supplementary material for: PRONTOX – proton therapy to reduce acute normal tissue toxicity in locally advanced non-small-cell lung carcinomas (NSCLC): study protocol for a randomised controlled trial
Source: Trials. 2016 Nov 15;17:543. doi: 10.1186/s13063-016-1679-4 (PMC5111266; doi:10.1186/s13063-016-1679-4)
Supplement: Additional file 2: Figure S1. — SPIRIT 2013 figure PRONTOX. (DOC 51 kb) [file 13063_2016_1679_MOESM2_ESM.doc]

|  | **STUDY PERIOD** | | | | | | | |
| --- | --- | --- | --- | --- | --- | --- | --- | --- |
|  | **Enrolment** | **Allocation** | **Post-allocation** | | | | | **Close-out** |
| **TIMEPOINT**** | ***-t_1_*** | **0** | ***1fx*** | ***33fx*** | ***Week 2,4,8, 10*** | ***Week 6*** | ***Week 12*** | ***t_x_*** |
| **ENROLMENT:** |  |  |  |  |  |  |  |  |
| **Eligibility screen** | X |  |  |  |  |  |  |  |
| **Informed consent** | X |  |  |  |  |  |  |  |
| ***Quality of life questionare*** | X |  |  |  |  |  |  |  |
| ***Assessment of tumor motion (4D CT)*** | X |  |  |  |  |  |  |  |
| **Allocation** |  | X |  |  |  |  |  |  |
| **INTERVENTIONS:** |  |  |  |  |  |  |  |  |
| ***Photon therapy*** |  |  |  |  |  |  |  |  |
| ***Proton therapy*** |  |  |  |  |  |  |  |  |
| **ASSESSMENTS:** |  |  |  |  |  |  |  |  |
| ***Clinical examination*** | X |  | X | X |  | X | X |  |
| ***CT scan*** | X |  | X | X |  | X | X. | X |
| ***Assessment of side effects*** |  |  | X | X | X | X | X | X |
| ***Quality of life questionnaire*** | X |  |  | X |  | X | X | X |
